# Effect of nitrogen application rate on yield, forage quality, and animal performance in a tropical pasture

**DOI:** 10.1038/s41598-019-44138-x

**Published:** 2019-05-20

**Authors:** Lutti M. Delevatti, Abmael S. Cardoso, Rondineli P. Barbero, Rhaony G. Leite, Eliéder P. Romanzini, Ana C. Ruggieri, Ricardo A. Reis

**Affiliations:** 1 UNESP - Univ Estadual Paulista, Departamento de Zootecnia, Faculdade de Ciências Agrárias e Veterinárias, 14884-900 Jaboticabal, SP Brazil; 2 UFRRJ - Univ Federal Rural do Rio de Janeiro, Departamento de Produção Animal, Instituto de Zootecnia, 23897-000 Seropédica, RJ Brazil; 3 Departamento de Ciências Exatas, Faculdade de Ciências Agrárias e Veterinárias, UNESP - Univ Estadual Paulista, 14884-900 Jaboticabal, SP Brazil

**Keywords:** Ecology, Plant sciences

## Abstract

A three-year-long field experiment was conducted in a continuous grazing system with a variable stocking rate to evaluate effects of increasing nitrogen levels in Marandu grass (*Brachiaria brizantha* Hochst ex A. Rich Stapf “marandu”) on herbage mass, forage accumulation rate (FAR), forage quality, stocking rate (SR), average daily gain (ADG), gain per hectare (GPH), and gain per kg of applied N. The experimental design was completely randomized with four treatments (control without application of N, and 90, 180, and 270 kg N ha^−1^ year^−1^) and three replicates (paddocks per treatment); nitrogen was applied in the form of urea. Herbage mass, crude protein (CP), FAR, SR, GPH, and the nitrogen nutrition index increased with increasing nitrogen level (P < 0.05), whereas the neutral detergent fibre (NDF), acid detergent fibre, and nitrogen usage efficiency decreased with increasing nitrogen level (P < 0.01). Crude protein was higher than 12% and NDF lower than 60% in all treatments. Nitrogen application rate affected ADG (P < 0.05) but did not fit any equation. The highest ADG was 90 kg N ha^−1^ year^−1^ (985 g animal^−1^ day^−1^). Increasing the nitrogen level is a promising way to improve Marandu grass production, nutritive value, and animal production.

## Introduction

Protein is a vital nutrient for human nutrition and animal meat is the main source of protein for the human population^1^. Beef is rich in high biological value proteins that are rich in essential amino acids and possess the highest quality rating among food sources^1^. The high quality of this source is primarily explained by the high chemical score and amino acid profiles of proteins obtained from animal sources^1^. However, the production of animal proteins requires millions of hectares of land and numerous resources^2^. Finding a way to enhance animal production, while saving resources and land, is among the main challenge for the animal science community^3^. Livestock productivity in tropical areas is low and reasons for this can be diverse, such as inadequate genetic resources, improper animal management, absence of pasture management, and low investment^4^. Most of the animal production in tropical areas is based on forage and inadequate grazing management can be responsible for insufficient yield in animal production systems^5^.

Forage production can be improved through fertilizer application, management of grazing, and control of weeds^6^. The effects of fertilizers on forage production are examined through plot experiments, which attempt to simulate the real production systems. Most studies on grazing strategies aim to improve forage production and animal performance and are based on pasture height. However, these studies typically investigate the effects of fertilizer use alone.

In 1950s, Brougham^7^ found that the highest net forage accumulation occurs when the grass achieves 95% of the incident light interception (IL). In continuous stocking grazing management, the pasture is maintained at near constant IL, which allows for a high photosynthetic rate and high herbage production^8^. Associating pasture management at 95% IL with nitrogen fertilizers could result in high forage production, nutritive value, and animal performance^9^. In this study, we aimed to quantify the effect of application of varying nitrogen doses on the forage yield, chemical composition, nitrogen usage efficiency (NUE), and animal performance in a tropical Marandu grass pasture. We hypothesized that combining grazing management with 95% IL and nitrogen application would: (1) increase the herbage mass (HM) and animal performance, (2) augment herbage quality, (3) enhance the ratio of absorbed and critical levels of nitrogen required by plants (nitrogen nutrition index – NNI), and (4) decrease the NUE.

## Results and Discussion

### Herbage yield and accumulation rate

Fertilization with nitrogen increased the HM immediately and consistently, as we did not observe effect of year of evaluation. In our study, herbage yield was affected by nitrogen application rate (P = 0.002), which increased linearly (P < 0.001) (Table 1). This difference in the herbage yield likely occurred because of the difference in tiller density, because the pasture height was the same^10^. Tiller density increases with increasing nitrogen application rates^10^. Our results were similar to those measured in a site 1200 km from our study (Cerrado region), and documented a value of 6098 kg DM ha^−1^ ^11^. However, the values obtained in this study were lower than the 7847 kg DM ha^−1^ reported in Kenya^12^, and the 9871 kg DM ha^−1^ reported in Brazil^13^.

**Table 1:** Herbage mass, morphological composition, forage allowance, and chemical composition of palisade grass pastures under fertilization with different doses of nitrogen. (Means of 3 years of evaluation, 4 evaluations per year, n = 36).

Item	Treatment (kg N ha^−1^)					Effect
	0	90	180	270	SEM	
**Herbage characteristics**						
Herbage mass (kg ha^−1^)	5798	6345	6436	6499	106	linear
Green leaves (g kg^−1^)	444.3	422.0	362.3	354.9	13.91	linear
Stem + sheath (g kg^−1^)	320.1	338.5	392.8	392.8	15.02	quadratic
Dead material (g kg^−1^)	235.6	239.7	244.8	252.3	12.23	ns
Herbage allowance (kg DM kg BW)	3.89	3.05	2.55	2.23	0.135	linear
Leaf allowance (kg DM kg BW)	1.78	1.29	0.95	0.79	0.094	linear
**Chemical composition**						
OM (g kg^−1^ DM)	916.1	918.4	919.0	916.5	1.03	ns
CP (g kg^−1^ DM)	113.6	135.5	150.9	167.6	5.41	linear
NDF (g kg^−1^ DM)	606.2	585.7	564.0	559.2	5.82	linear
ADF (g kg^−1^ DM)	298.1	287.3	278.7	276.2	4.05	quadratic
Lignin (g kg^−1^ DM)	39.5	35.6	38.5	37.7	1.03	ns
Ash (g kg^−1^ DM)	83.9	81.6	81.0	83.5	0.92	ns
DE (g kg^−1^ DM)	584.1	569.2	565.9	560.8	2.31	ns

Nitrogen (N), dry matter (DM), body weight (BW), organic matter (OM), crude protein (CP), neutral detergent fiber (NDF), acid detergent fiber (ADF), dry matter digestibility (DE), standard error of mean (SEM). ANOVA significance (Herbage mass P = 0.002; green leaves P = 0.003; stem + sheath P < 0.001; dead material P = 0.2599; CP P < 0.001; herbage allowance P < 0.049; leaf allowance P = 0.031, OM P = 0.027, NDF P < 0.001; ADF P < 0.001; lignin P > 0.05; ash P > 0.05; DE P = 0.35).

**Figure 1:**
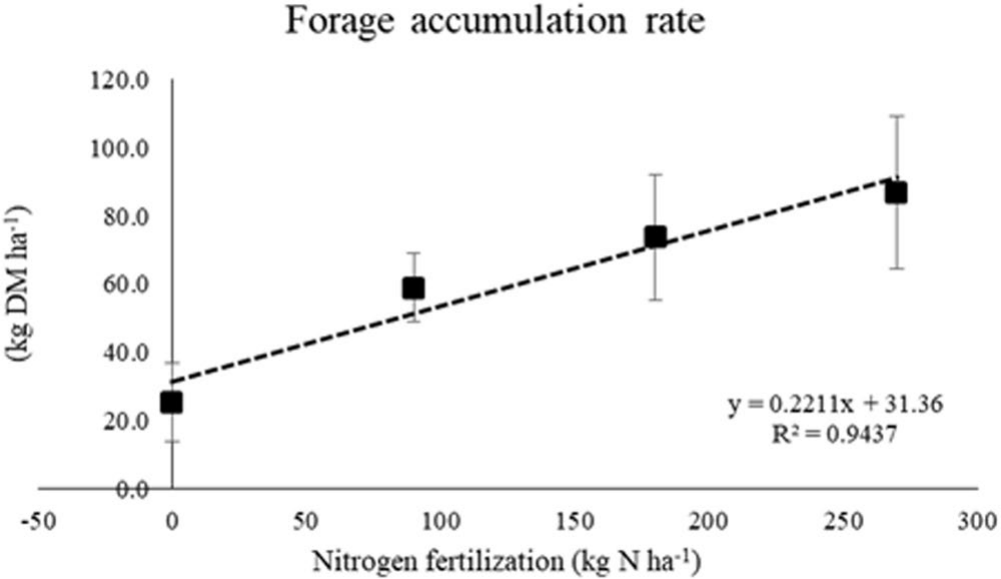
Forage accumulation rate (kg DM^−1^ day^−1^) of Marandu grass under fertilization with different doses of nitrogen. ANOVA (P = 0.043). The effect of nitrogen application was linear and positive. In the linear equation, f(x) is a function of N dose (x = kg N ha^−1^) and y = kg DM^−1^ day^−1^.

Forage accumulation rate (FAR) was affected by nitrogen application rates (P = 0.043). The effect of nitrogen application was linear (P < 0.01 (Fig. 1). These values were higher than those reported for a Cerrado region^11^, which was 63 kg DM ha^−1^ d^−1^, and lower than that reported in a similar region studied by our group^14^, being 106.4 kg DM ha^−1^ d^−1^. Our findings can be potentially explained by the grazing management that we adopted. Under different canopy heights at continuous stocking, the tiller size/density compensation results in small differences in the amount of leaves produced per unit area^15^. In our study, the sward heights were kept the same and the differences in FAR were caused by N supply variations. Because we managed the pasture with the goal of 95% IL, which occurs at 25 cm under our conditions^16^, we probably measured the maximal net forage accumulation, which occurs at 95% IL^8^. In tropical conditions, the values for nitrogen fertilizer application range from 300 to 400 kg N per ha^17^. This means that forage production can continue to increase in response to nitrogen application.

Increasing nitrogen application to crops can follow the Law of Diminishing Returns (Mitscherlich’s Law). Mitscherlich^18^ observed that with an increasing rate of fertilizer application, the crop yield increased, but at a decreasing rate. Here, we found that increase in the yield decreased per added unit of fertilizer. When 90 kg N ha^−1^ was applied, the FAR was augmented by a factor of 2.32, whereas after 180 kg N ha^−1^, it increased 1.25 times, and with an increase of 270 kg N ha^−1^, it augmented by a factor of 1.18. In Brazil, nitrogen fertilizers are expensive and the increase in the forage production resulting from the application of 270 kg N ha^−1^ may not result in higher profits for farmers.

Differences in the morphological composition of the sward with nitrogen application rates to grasslands occur because of increases in the leaf elongation rate, leaf area, changes in ontogenetic development, and decreased senescence rate^19^. However, when the canopy is managed as the same pasture, height differences in morphological composition are caused more by variations in climatic conditions^20^. Nitrogen application rates affected green leaves (Table 1). The green leaf content was lower than that reported when Marandu grasses were fertilized with nitrogen in rotational grazing^21^, and in line with that measured in a Cerrado biome^22^. The stem + sheath proportion varied with the nitrogen application rate (P < 0.01) quadratically and averaged approximately 350 g kg^−1^ DM (Table 1), being in line with the average value reported in Mata Atlantica^23^ and a similar ecosystem^24^. The amount of dead material was affected by nitrogen application rates (P < 0.05), and surprisingly, it showed a linear increase (Table 1). This was higher than the values reported in a similar climatic region^16^ and in rotational grazing^21^, and lower than that reported in a study in the Cerrado^11^. It also differed from the findings measured in a Mata Atlantica region^23^, where a reduction in dead material with increasing nitrogen application rate was observed. Increasing nitrogen application rate diminished the leaf life because of the higher plant growth^13^. The higher amount of dead material reported in this study at the highest nitrogen application rate could be a consequence of the higher stocking rate observed in this treatment. Trampling can affect forage utilization and may increase forage plant senescence^25^.

### Chemical composition

Nitrogen application affected the crude protein (CP) in the forage^22^. The amount of CP can increase with increasing nitrogen application rate. Augment is caused by increases in amino acids and protein synthesis^22^. In our study, CP increased linearly with DM (P < 0.01), from the lowest to the highest nitrogen application rate (Table 1), in agreement with findings for Marandu grass in a subtropical region^26^ and in the Cerrado biome^22^. In a review, the authors reported the CP of tropical grasses from 47 studies and calculated a mean of 82 g kg^−1^ DM with a range of 52 to 128 g kg^−1^ DM of CP^27^. The cultivar Marandu usually has less than 120 g kg^−1^ DM of CP in the total DM. Our results were higher values than those observed in other studies. This could be attributed to the sufficient inorganic N for luxury consumption (NNI > 1), as found in this study, and a combination of rainfall, precipitation, and temperature that allowed higher CP^28^. Nevertheless, in our experimental site, CP of approximately 150 g kg^−1^ DM has been reported in the last 16 years^24,29,30^. The tiller size/density compensation results in a high amount of leaves produced per unit area^15^ that may explain the CP values observed. Similar to the amounts found in our study, higher CP was measured in *Brachiaria* grass in Vietnam, which ranged from 131 to 170 g kg^−1^ DM^31^.

The neutral detergent fibre (NDF) concentration decreased linearly with increasing nitrogen application rate (P < 0.01) (Table 1). Our data agreed with the reduction in NDF concentration previously observed^22,32,33^. As observed in this study, the concentration of NDF diminished because of an increase in the CP and other soluble contents, which accumulated in the cell and cause dilution of the cell wall^34^. Results from a few other studies disagree with our findings; for example, they did not observe changes in NDF content caused by N rates^35^. These changes are associated with the epoch of evaluation mainly during the growing season. During the growing season, higher growing rates result in stem accumulation, thus NDF increases^15^. Nevertheless, the adoption of the criterion of herbage management at 95% LI reduces stem elongation because of grazing^13^.

Acid detergent fibre (ADF) decreased linearly as the nitrogen application rate was increased (P < 0.01), whereas the lignin content was unaffected (P > 0.05) (Table 1). These changes in ADF probably occurred because of the dilution of the cell wall as discussed above. Indeed, to increase CP content, the forage uses carbon to reduce inorganic N for protein synthesis rather than to produce structural carbohydrates^36^. ADF was lower than the values reported for *Brachiaria brizantha* in Vietnam^31^ and in the Cerrado biome^22^. A few other studies also disagreed with our findings^22,32^, wherein a reduction in the ADF content with N application was not observed. Increasing fertilization levels did not affect dry matter digestibility (P = 0.3539) that was within the range of 550–600 g kg^−1^ DM for *Brachiaria* cultivars^22,24,31^.

### NUE and nutrition index

The NUE is measured by the dry mass productivity per unit N taken from the soil^37^, and is influenced by the application rate, method, and timing of fertilizer application, as well as environmental and soil conditions^38^. In tropical pastures, the NUE values are typically low, at approximately 500 g kg^−1^ N applied. We found that NUE decreased linearly and was lower than the average found in tropical pastures (Table 2). These values were lower than the reported 427 g kg^−1^ applied for Marandu grass cultivated in a Cerrado region^22^. As N application rate increases, the NUE typically decreases, as observed in this study^39^.

**Table 2:** Apparent nitrogen recovery, nitrogen usage efficiency, and nitrogen nutrition index of palisade grass under fertilization with different doses of nitrogen.

Item	Treatment (kg N ha^−1^)					Effect
	0	90	180	270	SEM	
N recovery (kg N ha^−1^)	110.5	132.7	156.9	175.6	6.29	linear
NUE (g N used kg^−1^ N applied)	—	356.3	271.2	249.5	23.88	linear
NNI (dimensionless)	0.92	1.13	1.26	1.40	0.051	linear

(Means of 3 years of evaluation, 4 evaluations per year, n = 36). Nitrogen usage efficiency (NUE), nitrogen nutrition index (NNI), standard error of mean (SEM). ANOVA significance (N recovery P = 0.023; NUE P < 0.01; NNI P < 0.001).

Nitrogen recovery (N rec – kg N ha^−1^) from fertilizer application can vary according to the grass species, forage management, source of N, soil properties, and environmental factors^40^. The apparent nitrogen recovery (REC - % N applied recovered) from soil increased linearly with increasing nitrogen application rate, although the nitrogen recovered decreased linearly (Table 2). Both the amount of N rec from soil + fertilizer and the percentage of N from fertilizer were significantly affected by the treatments (P < 0.01). They decreased linearly (Table 2). Our results are in agreement with those measured for coast-cross, who found higher nitrogen agronomic efficiency when low rates of N were applied; that is, efficiency decreased with increasing nitrogen application rates^41^. In a Cerrado biome^22^ an N rec of 203.5 g kg^−1^ N applied was recorded, which is lower than our results. However, we found N rec values lower than those observed in a similar soil and climatic region^10^, which ranged from 450 to 920 g kg^−1^ N. These values were also lower than those verified in Europe for a range of forages, i.e. between 500 and 800 g kg^−1^ N^42^. The lower N rec found by us probably occurred because of the method of application (applied to surface), which results in higher nitrogen volatilization^43^. The highest flux values of ammonia were observed when urea was applied to the grassland soil surface^43^. Moreover, in tropical regions, the loss of N by volatilization can be higher because of high rainfall and higher temperatures^44^.

With respect to the nitrogen nutritional index (NNI), values < 1 indicated N deficiency, N = 1 was the critical level, and >1 was considered luxurious N consumption^45,46^. Nitrogen application rate increased the NNI values, paddocks without urea application presented a value of 0.92 and those receiving the highest urea application rate had a value of 1.40 (Table 2). Nitrogen deficiency was observed only in the control treatment. Nitrogen nutrition index means were >1 in the treatments with urea application, suggesting that luxurious N consumption occurred. Values of NNI above 1 can increase the production costs and potentially contribute to environmental pollution^47^. Our results differ from the NNI < 1 that was observed when 120 kg N ha^−1^ year^−1^ was applied in a *Brachiaria* pasture in Vietnam^31^. Nutritional status is an effective way to choose the best strategy for pasture fertilization. The NNI farms need to find the optimal N rate is based on the epoch and method of application to achieve an index equal 1. However, few studies reported a diagnosis of grazed pastures in tropical regions. Brazilian farms applied an average of 80 kg N ha^−1^ year^−1^ in pastures^47^. In the present study, the NNI for the dose of 90 kg N ha^−1^ year^−1^ was 1.13, probably the average rate of N used by the farms is close to the critical point of N nutrition according to the author of the index^45,46^ and represents the most beneficial rate of yield and expense for fertilizer. Our data suggest that applying relatively moderate amounts of N per year (approximately 200 kg^−1^ N) results in high yield and high forage quality.

### Animal performance

Average daily gain (ADG) was affected by the nitrogen level (P = 0.03) presenting a cubic effect. The highest ADG measured was 0.985 kg animal^−1^ day^−1^ (Table 3). The apparent small difference in the ADG among treatments was caused by the grazing management adopted, which was put-and-take^48^. In a put-and-take system, the stocking rate is adjusted to achieve the management target, which here is 25 cm pasture height; thereby, the best effect of nitrogen application on animal performance is observed in the gain per area. The ADG reported here is much higher than those previously observed: 0.510 kg animal^−1^ day^−1^ ^49^, 0.580 kg animal^−1^ day^−1^ ^50^, and 0.580 kg animal^−1^ day^−1^ ^51^. It was also higher than the national ADG of 0.27 kg animal^−1^ day^−1^ ^47^. However, it was lower than the value of 1.15 kg animal^−1^ day^−1^ measured in the same experimental area two years before our study^24^. The additional ADG can be attributed to animal supplementation (3 g supplement kg^−1^ BW). Higher ADG depends on dietary and non-dietary aspects that affect forage intake^52^. In this study, neither NDF nor CP limited the intake and N supply, which likely resulted in the higher ADG. Providing approximately 124 g CP kg^−1^ DM assures good availability of nitrogen for optimum animal metabolism, resulting in the best performance^27^. The CP reported in this study (>114 g kg^−1^ DM) probably resulted from a greater ruminal nitrogen supply, which increased ruminal digestion and microbial growth^27^. Non-nutritional factors affecting DM intake are herbage mass (kg DM ha^−1^), structural composition (e.g. ratio leaf/stem), and forage allowance (kg herbage mass available per kg body weight)^52^. Higher pasture dry matter intake and animal performance occurred when high pasture mass and allowance were provided.

**Table 3:** Effects of palisade grass fertilization with different doses of nitrogen on the performance of yearling bulls in the growing period.

Animal performance item	Treatment (kg N ha^−1^)					Effect
	0	90	180	270	SEM	
Stocking rate (AU ha^−1^)	3.37	4.64	5.81	6.55	0.234	linear
ADG (kg animal^−1^ day^−1^)	0.939	0.985	0.879	0.898	0.017	linear
GPH (kg BW ha^−1^)	514	769	848	967	51.3	linear
GPN (kg BW kg N applied^−1^)	—	2.84	1.86	1.68	0.361	linear

(Means of 3 years of evaluation, 4 evaluations per years, n = 36). Animal unit (AU) = 450 kg body weight (BW), dry matter (DM), organic matter (OM), crude protein (CP), average daily gain (ADG), gain per hectare (GPH), gain per kg N applied (GPN), and standard error of mean (SEM). ANOVA significance (Stocking rate P < 0.001; ADG P = 0.032; GPH P < 0.001; GPN P < 0.001).

A positive effect of increasing nitrogen levels was found on the stocking rate (SR), (LU = 450 kg ha^−1^; P < 0.001) (Table 3). Our stocking rate was higher than the values of 2.4 AU ha^−1^ reported for Marandu grass measured in a Mata Atlantica region^11^. In a Cerrado biome, a stocking rate of 4.0 LU ha^−1^ was measured during the summer^50^, which was similar to the value of 90 kg N ha^−1^ treatment in this study. Increase in the stocking rate occurred because of the increase in forage accumulation rate. These increases can be achieved using N fertilization and managing Marandu grass at 25 cm canopy height. The average Brazilian national stocking rate is 1.3 AU, which is much lower than the results of this study^53^.

The increase in nitrogen doses also increased the gain per area, which increased linearly during the 4 months of evaluation (rearing phase) (Table 3). They were also higher than the range of 470 to 778 kg BW ha^−1^ reported in a previous study in the same area evaluating different sward heights^24^. Nitrogen fertilization also increases yield, DM digestibility, and protein content of forage, leading to higher gain per area^54^.

An important index is the zootechnical efficiency of nitrogen usage (gain BW per kg N applied). In our study, the gain per kg nutrient applied decreased linearly (Table 3). A previous study calculated this index previously under Brazilian conditions^55^ and they reported a value of 2.44 kg BW kg^−1^ N from three studies with Marandu grass, that was within the range reported in the present study.

### Implications

Our results show that under continuous grazing, Marandu grass fertilized with 90 kg N ha^−1^ year^−1^ and managed at 25 cm height, which represents 95% IL in our soil and climatic conditions, produces high forage yield with high quality. The average Brazilian national stocking rate is 1.3 AU^53^ and the national ADG is 0.27 kg animal^−1^ day^−1^ ^47^. Our findings show that it is possible to increase the national average of stocking rate by four times and the productivity by three times, by solely feeding the animals with Marandu grass and a mineral mixture. Our results are applicable to the livestock region called the Brazil central region. The region is similar to the tropical regions with savannas, Oxisols, and seasonality in the rainfall distribution. Optimising pasture management in pasture-based beef production systems in tropical regions represents a sustainable method to save land and sustainably intensify production. Even though there was an observed increase in productivity parameters with increasing nitrogen doses, fertilizer use and dose must be adopted according to the system’s financial goals and market background^29^.

Nitrogen fertilization has been overused in several countries, resulting in high production costs and environmental impacts, such as increases in greenhouse gas emissions, pollution of water, and loss of biodiversity^56^. In this study, we report a high forage yield and high quality using a relatively low nitrogen application. Once nitrogen nutrition in all nitrogen application rates are in the margin of luxury consumption, the dose of 90 kg N ha^−1^ year^−1^ can be applied in Marandu grass in tropical clays soils. With this dose, it is possible to increase 0.3 kg BW gain animal^−1^ day^−1^ and doubled herbage mass production. In Brazil, nitrogen fertilizers are expensive and the increase in the forage resulting from the application of 270 kg N ha^−1^ may not result in a higher profit for farmers.

With respect to greenhouse gas emissions, different scenarios of beef cattle production in Brazil were analysed^47^. They calculated that, in intensive systems based on grasslands, the carbon footprint is four times lower than the national average, and it is possible to increase land efficiency by three times. In the intensive scenario studied, they considered an ADG of 0.75 kg animal^−1^ day^−1^ and stocking rate of 3 AU^47^. In the present study, we report an ADG of 0.93 kg animal^−1^ day^−1^ and stocking rate of 6 AU, indicating that is possible to increase land use efficiency even more, while simultaneously diminishing the carbon footprint of beef cattle in Brazil.

Based on our results, we recommend that the farms and consultants adopt the strategies of management used in this study, namely, continuous grazing with the put-and-take method, sward at 95% IL, 50% of grazing efficiency, and moderate nitrogen fertilization (180 kg N ha^−1^ year^−1^) to achieve higher animal productivity. Once ADG is improved, the age of slaughter can be reduced and our strategy of fertilization can indirectly contribute to the reduction of greenhouse gas emissions. In Brazil, the average age of slaughter is 3 years, which can be reduced to 2 years with moderate intensification. The increases in the gain per area may also contribute to saving land once less area is required to produce the same amount of meat. However, we stress that our results are applicable to intensive systems with continuous grazing, which were rigorous in all management aspects, with the goal of to achieving the optimal grazing pressure postulated by Mott without pasture degradation^57^.

## Conclusions

Application of relatively low nitrogen doses results in high yield and high quality of Marandu grass under continuous stock. The herbage, forage accumulation rate, and nutritive value increased linearly with nitrogen application rate. Our data are applicable to that of tropical areas with Oxisol, seasonal rainfall (wet summer), Marandu palisade-grass, and Nellore cattle during the rearing phase.

The apparent nitrogen recovery was inversely proportional to the nitrogen application rates. The critical N increased with increasing nitrogen level and the NNI showed that the N application was enough to supply the critical N level for Marandu grass nutrition.

Average daily gain and gain per area increased with the nitrogen dose and were higher than the previously published values for young Nellore bulls reared solely on tropical grasses.

The grazing management adopted here with relatively low N application rates can be used for the sustainable intensification of pasture-based beef in tropical areas. This could potentially improve land use efficiency, reduce pressure to open new areas, release areas for crops, and indirectly reduce greenhouse gas emissions. The impact of the Marandu grass management during the growing season (from November to April) studied by us was immediate and was maintained throughout three years of evaluation.

In Brazil, nitrogen fertilizers are expensive and the increase in the forage resulting from application of 270 kg N ha^−1^ may not result in a higher profit for the farmers. Our data suggest that applying relatively moderate amounts of N per year (approximately 180 kg^−1^ N) results in high yield and high forage quality.

## Methods

### Experimental area and design

A three-year-long experiment was conducted in the Forage and Grasslands sector of São Paulo State University, “Julio de Mesquita Filho” (UNESP) (Jaboticabal, São Paulo, Brazil), during the forage growing season (December to April) of 2014/2015, 2015/2016, and 2016/2017. Climate of this region is classified as the subtropical humid type, with wet summers and dry winters. The mean annual rainfall is 1424 mm, mean air temperature is 22.3 °C, and the soil is a Rhodic Ferralsol^58^ derived from basalt. The pasture was established in 2001 with *Brachiaria brizantha* (Hochst ex A. Rich) Stapf Marandu (Marandu grass). Soil chemical and physical characteristics are presented in Table 4.

**Table 4:** Soil chemical composition and texture in the Marandu grass pasture paddocks under fertilization with different doses of nitrogen.

Treatments (kg N ha^−1^)	Soil chemical composition								Soil texture^*^			SBD^5^
	pH	SOM	P	K	Ca	Mg	H + Al	V	Clay	Lime	Sand	
		g dm^−3^	mg dm^−3^	mmolc dm^−3^				%	g kg^−1^			g cm^−3^
0	5.2	29	8	12	15	9	22	55	269	98	633	1.59
90	5.3	29	11	2.2	20	11	20	63	291	86	623	1.57
180	5.1	28	9	2.7	18	9	25	56	312	107	581	1.61
270	5.1	16	11	2.7	16	9	24	55	284	94	622	1.58

(pH) Soil pH in CaCl_2_; (SOM) soil organic matter; (V) percent of base saturation; (SBD) soil bulk density. *Soil texture was sandy-clay-loam.

The experimental design was a completely randomized design, with four treatments (0, 90, 180, and 270 kg N ha^−1^) using urea fertilizer, which were applied in a split design during 3 fertilizations per growing season, and 3 replicates per treatment, totalling 12 paddocks (experimental units). The paddock areas were 1.3 ha, 1 ha, 0.7 ha, and 0.5 ha for the treatments 0, 90, 180, and 270 kg N ha^−1^, respectively. The experimental area included a reserve area of 3 ha for the spare animals.

### Animal and grazing management

Animals involved in this study were cared for according to the rules of the São Paulo State University Animal Care and Use Committee and the National Council of Animal Experimentation Control. The committee reviewed and approved the experiment and all procedures carried out in the study (Certificate number 12703/15). Seventy-two young Nellore bulls (*Bos indicus*) in each year with an initial BW (mean ± SD) of 352 ± 5 kg, 334 ± 2 kg, and 315 ± 6 kg were utilized to measure animal productivity in the first, second, and third experimental year, respectively. The bulls were identified, weighed, and randomly distributed in groups of six bulls per paddock balanced for BW. The remaining animals were used to maintain a pre-determined grazing height, using the put-and-take methodology^48^.

The pasture was managed using the put-and-take methodology under continuous stocking. The pasture height of 25 cm was chosen because at this height, the canopy intercepted 95% of the light under our experimental conditions. At this IL, it is possible to achieve the maximal net forage accumulation^6^, which may result in high animal performance^13,24,59^. During the experimental period, the bulls were weighed every 28 days (without fasting) to adjust the stocking rate to the pasture height. During the experimental period, the animal diet was solely Marandu grass and mineral salt supplementation.

### Forage samples

During the experimental period, the grazing height was measured every 28 days. Eighty measurements per hectare were obtained using a ruler graduated in centimetres at the curvature of the upper leaves. Herbage mass was estimated using eight samples collected per cut^24^ at the average pasture height points of the paddock (approximately 5.0 cm of residue) using a 0.25 m^2^ circular frame. Forage samples were separated into green leaves, stem + sheath, and dead material, and then dried in an oven (55 ± 5 °C for 72 h), following which, they were weighed to calculate the forage dry mass per hectare. To evaluate the herbage yield, samples were collected as described above.

To determinate the forage composition, samples were collected from 20 points per hectare via the grazing-simulation method^24^, with approximately 200 g of fresh matter harvested per sample. Samples were dried in a forced-air oven (55 ± 5 °C, for 72 h), ground in a mill (Thomas-Wiley Laboratory Mill Model 4, H. Thomas Co.), and taken to the laboratory for analyses.

The cumulative forage dry mass was evaluated using exclusion cages (1 m^2^) and the triple pairing technique^60^. In this technique, forage samples are collected at 5 cm height from the ground and the cages are used to isolate the spots accrued because of the presence of young bulls under continuous grazing in the experimental area. On the first day of placement of the cages, two areas with similar dry matter of Marandu grass were selected by visual criteria, representing the paddock condition. To estimate the initial forage mass of Marandu grass inside the cage, we placed the cage in an area, and collected samples from a 1 m^2^ area both inside and outside the cage (paired samples) at 5 cm height from the ground. After 28 days of cage placement, forage samples were collected again to obtain herbage accumulation. The samples were then weighed to obtain the fresh mass, and subsamples of approximately 200 g were separated and dried in a forced-air ventilation oven at 55 °C for 72 h to estimate the dry weight and calculate the dry mass (kg ha^−1^).

Forage accumulation rate (kg ha^−1^ day^−1^) was calculated by dividing the cumulative forage dry mass by the number of days between evaluations (28 d). Cumulative forage dry mass was obtained by the difference between the forage dry mass of the samples collected from the interior of the cage on the date of sampling and the forage dry mass of the sample outside the cage (paired sample) on the date of the previous sampling.

### Chemical composition

Dry matter (DM), organic matter (OM), and ash were estimated following the procedures described in AOAC^61^ (AOAC 934.01 for DM, AOAC 942.05 for OM, and AOAC 942.05 for ash). Crude protein content was estimated using a LECO® FP 528 device (Leco Corporation, Michigan, USA). Neutral detergent fibre (NDF), acid detergent fibre (ADF), and acid detergent lignin (ADL) were determined using the procedures described by ANKOM Technology^62–64^.

### NUE and nutrition index

Apparent N recovery (REC) by the forage was estimated^45,46^ based on N uptake of the fertilized treatments and unfertilized control as follows:

$${\rm{REC}}( \% )=\{({{\rm{U}}}_{{\rm{f}}}-{{\rm{U}}}_{{\rm{o}}})/{{\rm{N}}}_{{\rm{f}}}\}$$

where N_f_ = fertilizer-N rate (kg ha^−1^) and U_f_ = N uptake (kg ha^−1^) when N_f_ is given, and U_o_ = N uptake (kilograms per hectare) in non-fertilized paddocks.

Apparent efficiency of absorbed N (NUE) by the plants was calculated^45,46^ as follows:

$$\mathrm{NUE}(\mathrm{kg}\,{{\rm{kg}}}^{-1}{\rm{N}})=\{({\rm{TFM}})/{\rm{Nc}}\}$$

where TFM = total forage mass of the treatment in kg and Nc = nitrogen uptake by the pastures in kg.

Nutrient yields were calculated by multiplying the DM yield by nutrient concentration in each sampling period. Nitrogen nutrition index (NNI) of Marandu grass was calculated^45,46^ as:

$${\rm{NNI}}=({\rm{Na}}/{\rm{Nc}})$$

where Nc = aW^−b^, where Na is the actual N concentration, Nc is the critical N concentration, W is the DM yield (Mg ha^−1^), and a and b are species-specific constants for C_4_ perennial grasses (3.6 and 0.34, respectively; refs^45,46,65^). Luxury N consumption was assumed for NNI values > 1^45,46^.

### Animal production

Bulls were weighed at the beginning (0 days) of the experiment, at the end of the adaptation period of each year, and at the end of the experimental period, after fasting for 12 h prior to each weighing event. The bulls were also weighed every 28 days (without fasting), to adjust the stocking rate to the maintain pasture height. Animal performance variables were calculated as follows^24^:

$${\rm{Body}}\,{\rm{weight}}\,\mathrm{gain} \mbox{-} \mathrm{BW}\,{\rm{gain}}({\rm{kg}})=({}_{{\rm{final}}}{\rm{BW}}-{}_{{\rm{initial}}}{\rm{BW}});$$

$${\rm{Average}}\,{\rm{daily}}\,\mathrm{gain} \mbox{-} \mathrm{ADG}(\mathrm{kg}\,{{\rm{day}}}^{-1})=({\rm{BW}}\,{\rm{gain}}({\rm{kg}})/{\rm{days}});$$

$${\rm{Gain}}\,{\rm{per}}\,\mathrm{area} \mbox{-} \mathrm{GPH}=({\rm{ADG}}\times {\rm{number}}\,{\rm{of}}\,{\rm{animals}}\,{\rm{per}}\,{\rm{days}}\times {\rm{experimental}}\,{\rm{period}}({\rm{days}}))/\mathrm{area}\,({\rm{ha}}))$$

where the number of animal days (animal day^−1^ ha^−1^) was calculated by dividing animal stock by the mean weight of “testers”. Animal stock was obtained by the sum of weights of all animals present in each paddock divided by the area of the paddock (kg BW ha^−1^);

$${\rm{Gain}}\,{\rm{per}}\,{\rm{kg}}\,{\rm{N}}\,{\rm{applied}} \mbox{-} \mathrm{GPN}(\mathrm{kg}\,{\rm{gain}}\,{{\rm{kg}}}^{-1}{\rm{N}}\,\mathrm{applied})=({\rm{GPH}}/{\rm{kg}}\,{\rm{of}}\,{\rm{N}}\,{\rm{applied}});$$

$${\rm{Stocking}}\,{\rm{rate}}\,{\rm{in}}\,{\rm{animal}}\,{\rm{unit}}({\rm{AU}}=450\,{\rm{kg}}\,{\rm{BW}})/{\rm{ha}}=(\sum \,{\rm{BWmean}}/450)/{\rm{area}}({\rm{ha}});$$

$${\rm{Herbage}}\,{\rm{mass}}\,{\rm{allowance}}({\rm{kg}}\,{\rm{DM}}/{\rm{kg}}\,{\rm{BW}})=({\rm{kg}}\,{\rm{DM}}/{\rm{ha}})/(\sum {\rm{BWmean}}/{\rm{area}}({\rm{ha}})).$$

### Statistical analysis

Data were tested for normality and equal variance using the Shapiro-Wilk normality test and Bartlett test of homogeneity of variances, respectively. Data were analysed using the LME procedure of R (package NLME, R core team^66^), and the statistical model included nitrogen level and year as fixed effects, whereas paddock or animal was the random effect. All variables were analysed as repeated measures. The best covariance structure used for repeated-measures analyses was chosen as the one that achieved the lowest corrected Akaike and Bayesian information. Significant effects for treatment were declared at P < 0.05. When a significant effect was found, orthogonal polynomial contrasts were performed to assess the effect of nitrogen dose on the variables.

## Data Availability

Data will be made available upon request for authors.
